# Public Health Messaging During Extreme Smoke Events: Are We Hitting the Mark?

**DOI:** 10.3389/fpubh.2020.00465

**Published:** 2020-09-02

**Authors:** M. Therese Marfori, Sharon L. Campbell, Kate Garvey, Scott McKeown, Mark Veitch, Amanda J. Wheeler, Nicolas Borchers-Arriagada, Fay H. Johnston

**Affiliations:** ^1^Environmental Health Group, Menzies Institute for Medical Research, University of Tasmania, Hobart, TAS, Australia; ^2^Public Health Services, Department of Health, Tasmanian Government, Hobart, TAS, Australia; ^3^Mary MacKillop Institute for Health Research, Australian Catholic University, Melbourne, VIC, Australia

**Keywords:** air quality, PM2.5, fire, risk communication, smoke, public health, social media

## Abstract

**Background:** Emergency services working to protect communities from harm during wildfires aim to provide regular public advisories on the hazards from fire and smoke. However, there are few studies evaluating the success of public health communications regarding the management of smoke exposure. We explored the responses to smoke-related health advisories of people living in a severely smoke-affected region during extensive wildfires in Tasmania, Australia early in 2019. We also evaluated the acceptability of portable high efficiency particle air (HEPA) cleaners used in study participant's homes during the smoky period.

**Methods:** We conducted semi-structured interviews with 24 households in the Huon Valley region of Tasmania following a severe smoke episode. These households were initially recruited into a HEPA cleaner study. Interviews were recorded, transcribed, and analyzed for common themes using an inductive framework approach.

**Results:** Public health messaging during the 2019 wildfire event in Tasmania was widely shared and understood, with social media playing a central role. However, some participants expressed concerns about the timeliness and effectiveness of the recommended interventions, and some would have appreciated more detailed information about the health risks from smoke. Public messages and actions to protect households from wildfire threat were, at times, contradictory or dominated in coverage over the smoke messaging, and many participants were conflicted with the multiple public messages and action relating to the more serious perceived threat from the fire.

**Conclusions:** Public messaging about smoke and health should continue to use multiple avenues of communication, with a focus on simple messages provided through social media. Messaging about the smoke hazard should be available from a trusted central source regarding all aspects of the wildfire emergency, with links to more detailed information including local air quality data alongside interpretation of the associated health risks.

## Introduction

Changing global environmental conditions have increased the frequency, intensity and spread of wildfires with substantial implications for human health (1). Destructive wildfires close to human communities and infrastructure can directly cause considerable economic losses and claim human lives, while the associated air pollution adversely affects human health (2, 3). Wildfire smoke contains large amounts of fine particulate matter which cause a wide range of health problems, from upper respiratory tract irritation to exacerbations of cardiac, respiratory and other chronic conditions (4–6). This leads to increased morbidity and mortality, and a significant economic burden to affected populations (7–10). Specific populations are more susceptible to adverse health effects from wildfire smoke, such as those with pre-existing respiratory or cardiovascular disease, children, pregnant women, and the elderly (5, 11, 12).

Wildfire smoke presents some challenges for public health management because exposures can be extreme, while emissions are not generally amenable to control and can affect large geographic areas and populations (13, 14). Standard public health strategies focus on risk assessment and public communications to provide recommendations for mitigating the risk of adverse health impacts from smoke (15). Public health communication or messaging seeks to improve understanding of individual risk and direct them to appropriate responses. The messages are often promulgated through traditional and social media accounts managed by agencies. They generally center on communicating who is at higher risk from smoke and specific actions they can take to reduce exposure to smoke, such as: staying indoors, reducing outdoor activities, the appropriate use of masks and air cleaners, relocation, or to manage health conditions affected by smoke, such as asthma (16–18).

However, there is a paucity of information regarding the effectiveness of public health messaging on smoke during wildfire smoke events (5, 19). Few published studies have evaluated risk communication strategies in depth, although some community surveys after the pollution episode have been conducted to measure the recall of public health messaging and behavior change related to public health advisories (19). During emergency responses, there can be extensive public communications, including emergency evacuations and warnings for wildfires, as well as those relating to associated hazards, such as poor air quality from smoke. Effective risk communication is challenging but when done well can engender trust and support individuals to make informed decisions (20).

**Figure 1:**
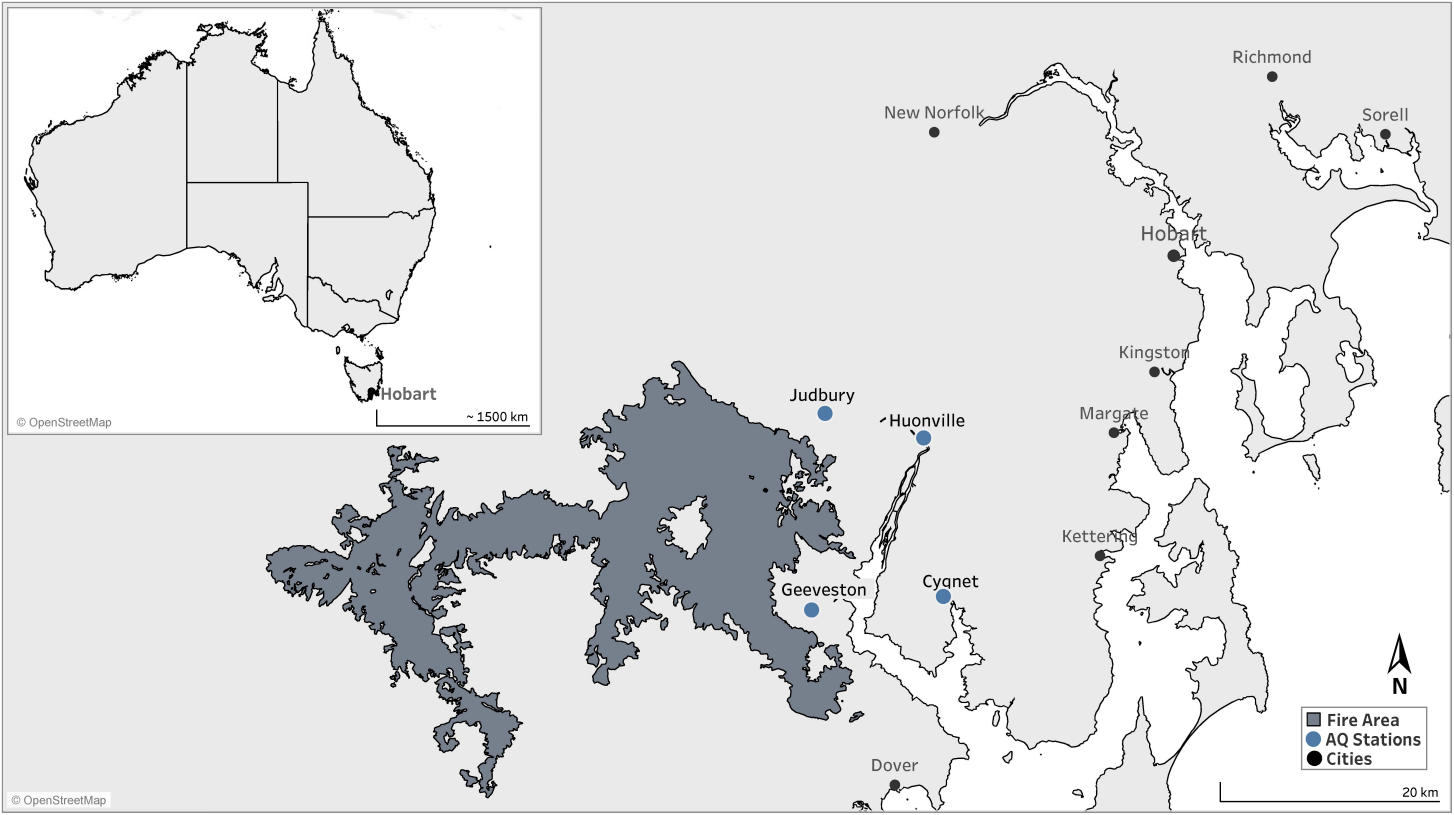
Map of the southern region of Tasmania, Australia. The proximal firegrounds during the 2019 wildfires are shaded.

**Figure 2:**
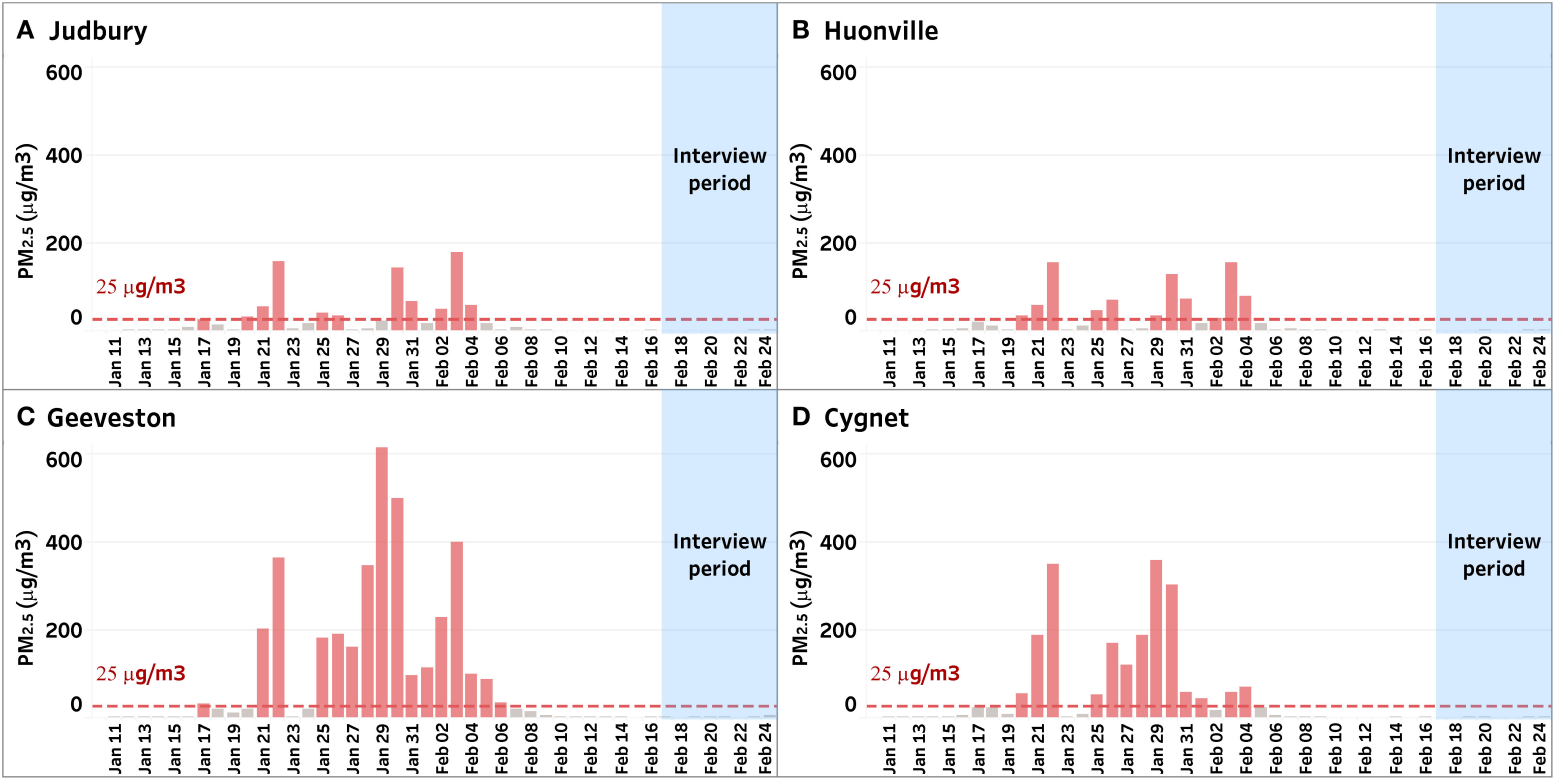
Daily average mass of particulate matter <2.5 microns in diameter per cubic meter of air (PM2.5) from four locations, Judbury **(A)**, Huonville **(B)**, Geeveston **(C)**, and Cygnet **(D)**, in the Huon Valley during the wildfire period from 10 January 2019 to 24 February 2019. Most participants in this study lived in or near the town of Cygnet **(D)**.

We conducted a qualitative study about perceptions of public health messaging relating to smoke during a sustained wildfire episode in early 2019, the height of the austral summer. Hundreds of fires in densely vegetated parts of the island state of Tasmania were ignited by dry lightning, many of which coalesced in difficult terrain for firefighting. The Huon Valley region, south of the capital city of Hobart, was affected by both direct attack from fire fronts prompting the evacuation of some areas, and by more than a month of fluctuating and often extremely poor air quality (Figures 1, 2). During the worst period of air pollution, public health authorities advised residents at higher risk of adverse health impacts from smoke to consider relocation to places with better air quality. Authorities also established a smoke refuge, with transport provided for residents who were unable to find their own alternative accommodation.

We received expedited ethical approval to conduct a rapid study during the public health emergency. In a separate study, we evaluated the effectiveness of portable high efficiency particle air (HEPA) cleaners to improve residential indoor air quality during extreme episodes of air pollution. We also conducted a simultaneous qualitative evaluation of perceptions of the public health messaging regarding wildfire smoke and the perceived usefulness of the air cleaners among study participants. Here we present results from the qualitative component of this study in which we aimed to understand (1) the level of concern about the impacts of smoke on well-being, (2) how information about smoke and health was received and understood, (3) if public health information influenced individual actions and behavior, and (4) the acceptability of using portable HEPA cleaners for managing poor indoor air quality during the wildfires.

## Methods

### Participant Selection and Recruitment

The study was constrained by the ongoing emergency and the need for a rapid response. As such it was small in scale and the initial target number of participants was between 25 and 30. Residents from the Huon Valley were invited to join the study if (1) they were available for a 3 weeks period for continual indoor and outdoor air quality monitoring with intermittent use of a HEPA cleaner, (2) did not have household members who smoked as this would have increased the complexity of interpreting the indoor air quality measurements for the HEPA evaluation study, and (3) lived in parts of the valley that were not at high risk of direct attack from the wildfire front. This excluded much of the western side of the valley where emergency warnings and evacuation centers were already in place. As a part of the process for obtaining written consent it was explained that all public health and emergency warnings should be followed even if this meant abandoning participation in the study. In this way, participation in the research project did not influence any participants' decision to stay in their home or move to places less affected by either the wildfires or the associated smoke.

Participants were recruited through multiple channels including advertising via news media, including radio and newspaper, and through the social media feeds of Public Health Services (Department of Health, Tasmania) and regional community groups. We also directly invited current users of the smartphone app *AirRater*, which provides real-time air quality information, who lived in the target area and had previously agreed to be contacted about potential research participation (21).

### Data Collection and Analysis

Data were collected via face-to-face semi-structured interviews in the participants homes on the final visit for the air cleaner study. These interviews were conducted between 17/02/2019 and 24/02/2019, ~2–3 weeks after the fire. We used an interview template with four open-ended questions relating to each of our main aims: “Were you worried about the health impacts from the bushfire smoke?”, “Do you remember receiving any health advice about the smoke?”, “Did you do anything different because of the information you heard about the smoke?”, and “Do you think the air cleaners were useful in improving your indoor air quality.” Each question was followed with in-depth discussion and follow up questions to clarify their responses. For example, if the participant discussed receiving information via *Facebook*, they were specifically asked for more details about the information they received from that source. Additionally, we sought comments on common themes broached by earlier participants. For example, several participants discussed future use of HEPA cleaners for situations other than wildfire smoke, and this was explored with subsequent participants.

All interviews were conducted by the same individual, then recorded and transcribed verbatim for analysis. We conducted an inductive thematic analysis of the qualitative data using the framework approach (22). This approach has been used in qualitative research since the 1980s and is commonly used for thematic analysis of semi-structured interview transcripts (22). It consists of five stages: familiarization of data, identifying a thematic framework, indexing the data by applying the thematic framework, charting or rearranging the data, and mapping and interpreting the data. Each transcript was repeatedly read with points related to research questions highlighted and provisionally coded. Key themes and codes were generated to form the thematic framework. QSR International's NVivo 12 software was used to assist with the indexing and charting stages of the analysis (23).

### Ethics

This study was approved by the Human Research Ethics Committee of the University of Tasmania (Ethics Ref No: H0017896).

## Results

Residents from 24 households were interviewed, 22 face-to-face in their homes and two via telephone for participants with time constraints on the day of the final home visit. Participants were mostly female (N 18/24) with life stages ranging from being parents of young children to retirees. Several later disclosed that they had a health condition affected by smoke, most commonly asthma, while others were either pregnant or had children <5 years of age. All of these fit the criteria for being at higher risk from exposure to air pollution from wildfire smoke and in the intended target group for public health messaging.

Several key themes emerged for each discussion topic and these are presented below.

### Personal Concerns Living Through a Smoke Event

Participants almost universally commented about experiencing negative physical, social and psychological impacts from living in smoke-affected areas over a prolonged period. The conditions were described with words, such as *depressing, stifling*, and the community becoming *like a ghost town*.

“*It was debilitating. Apart from the panic, the not being able to breathe, it was depressing. It made me really depressed. It felt like I was in purgatory”*

“*… it made you feel a little sick, it was really unpleasant. I got headaches, really bad headaches.”*

“*It's not just physical, it's also an emotional thing, being surrounded by the smoke all the time.”*

Others were more concerned by the threat of nearby wildfires than the smoke.

“*If you're only talking about the smoke, to us, where we are located it's a minor issue. The fire and possible embers, that's more worrying.”*

“*I was more worried that the fire might jump the river and come here, and I was in no way prepared to defend my house, so I just left. I wasn't so much worried—well actually, no, I was worried about the smoke because there was a lot of it.”*

Others expressed the tension of living through poor air quality while feeling that they and others could not fully acknowledge their smoke-related difficulties because of the concurrent fire emergency.

“*(Our town) was hardly mentioned in the whole fire drama. Which is fine, we're on the other side of the river, we were completely safe. But we weren't from the smoke. We were absolutely in the thick of it. And that wasn't mentioned.”*

Overall, the smoke event caused notable negative physical and mental impacts, but the concurrent wildfire event generally overshadowed concerns about the associated smoke risk for individuals who were smoke-affected and in media reporting of the event.

### Sources of Information for Smoke and Health

All participants recalled receiving information related to health impacts of smoke with a wide range of sources described. These included media releases, web-based information and social media posts from public health and emergency services, informal sources, such as friends and colleagues, and information passed around social media networks by individuals, community groups, and agencies. The Australian Broadcasting Corporation (ABC) is a public broadcasting station and is the official national emergency broadcaster. Tasmanian Fire Service promotes listening to ABC coverage during wildfire events, and it was a common source of messaging among participants. Social media was also a commonly reported source of both official and informal information and was the forum in which issues relating to smoke were able to gain salience amongst wildfire dominated news.

“*Yeah, I mean it [smoke information] was everywhere. No matter where I looked on Facebook, it was shared and posted and yeah, it was all over online, the radio stations were having it as well. It was definitely plenty of info out there.”*

“*I think the information that came out of it was brilliant. As I said, there's just so much info coming out every day from different media sources, so, and ‘cause everyone was watching on Facebook and ABC radio as well. Yeah as I said, I just got so much info every day. I kept leaning on it for what's going on around the place”*

A challenge was discerning which information to rely on. Almost all participants who used social media indicated there was a degree of mistrust of some the information shared. There was also concern that the social media platforms amplified disproportionate reactions to the smoke:

“*The trouble of social media is that it's a small-town mentality … so, you get one person saying, ‘it's terrible’ and then another person says, ‘it's terrible’ and then they start to panic.”*

“*A lot of scare tactics. They had a few graphs of what it [air pollution] was like in Beijing. One day I think in … this area, it was 50 times more or something. Something phenomenal. Whether or not that's true or not, I don't know. That's the other thing. You can't believe everything you see on social media … You don't know if it's been altered. You don't know, that's the thing.”*

“*I didn't take any notice of what was on Facebook, apart from what the councils put up in terms of their briefings. And I watched those twice a day … I think they were almost the best information.”*

“*I'd say on social media there's more misinformation than anything else. You know, it got a bit annoying … You just learn to ignore it.”*

In contrast, information shared by government agencies or trusted community members were valued and viewed as an accessible avenue to reliable information:

“*But I think the challenge these days with emergencies is that people go to Facebook. And read random things that random people have written, which are often incorrect … It was great to see TFS [Tasmania Fire Service] doing live video feeds, so it's still accessible through those mediums but credible information gets lost.”*

“*And in the [community] group, I think we've got a [general practitioner] and he explained in great detail—he's active quite a lot, actually. If there's community issues, he posts, he takes great effort to explain or explain the message with this health issue.”*

A key strength of receiving information via social media was the ability to peruse and process the information at one's own pace, and filter the relevant information in a low-stress environment:

“*I think I went along to one of the fire meetings organized by the fire department in Cygnet, and I listened online to a few livestreams when it was in Huonville, and they must've mentioned it [smoke] as well. But I can't really recall—I think they must have mentioned it—but I can't really recall in great detail, but I think Facebook made a greater impact. I can recall it better than the meetings, probably because the meetings are loaded with more information, and you are so focused on hearing what the fires are doing, and the health impacts from the smoke seems like a secondary concern.”*

An emergent theme was that active information-seeking was generally in pursuit of wildfire updates while exposure to smoke information was more incidental. Consequently, some pockets of the community may have missed messaging about smoke, particularly for those who were not well-connected or those who did not use social media.

“*I wasn't really seeking out anything in regards to the smoke. More just seeking out things do to with the fire.”*

“*I was going straight to the TFS, AlertTas on Twitter, and things like that. But it didn't cover the smoke impact it was more about tracking the fire.”*

“*I don't have access to TV, I only listen to the radio when I'm in a vehicle, and I hadn't seen any posters or anything. It [smoke information] was more about neighbors and things passing on random information.”*

“*… But [if] there's actually a stall about the smoke hazards where people frequent, and you could talk to someone face-to-face, well, it would reach me.”*

On follow up questioning regarding if public health information was easy to find, a couple acknowledged the ease of information sharing through social media and the potential implications on populations who do not use social media:

“A: *Yeah*

B: *Yeah, with the help of Facebook*

A: *I don't know if you were an older person who didn't have Facebook, maybe not? You might have found it harder.”*

Participants indicated there were many online avenues to official information related to wildfires and smoke; however, the necessity to check multiple different sources was burdensome. Several advocated for a central resource which provided clear links to the different information sources for both wildfires and smoke.

“*You had to seek them out a bit. It was a bit annoying because there should've been one website you could've gone for everything for people under stressful situation—you had to look up several different websites, different kinds of information.”*

“*We were on the TasFire Alerts page a lot, and I'm thinking maybe a link on that website would've been helpful? I would've used that.”*

### Perceptions of the Public Health Messaging on Smoke

Several themes emerged relating to the content of the public health messaging. All participants were able to recall much of the simple and direct messages disseminated by the public health response agency, which were almost entirely focused on groups of people more vulnerable to health harms from smoke. However, many expressed a desire for more nuanced and detailed information about the likely health harms from smoke exposure for all members of the community, and how the risks relate to the measured air quality. Many also commented that the information, especially the advice to relocate, was not timely as it came toward the end of the smoke affected period, and that some of the advice was not practical to implement. Others noted competing messages about managing the risks from fire and from smoke, such as simultaneous advice to go outside to reduce fuels near their house to reduce the fire risk, and to avoid doing exertional activity and stay inside to reduce the smoke risk.

#### Simple and Understandable Main Messages

All participants were able to recall some components of public health messages related to smoke, particularly the targeted populations who were advised to consider relocating from smoke-affected areas:

“*They were talking about how people who were asthmatic needed to leave the area … If you had any breathing problems or respiratory problems, and to leave if you can. Or seek medical help.”*

“*They basically were talking about the smoke refuge in Kingston, and if anyone had lung issues or had young children, they could evacuate to that smoke center.”*

“*Mostly if you're too concerned, or if it was impacting your health, to leave.”*

Most households (17 of 24) indicated that the messages surrounding actions to take were easily understood:

“*It was put out there very well. It was pretty straightforward”*

“*It was pitched at quite a simple level of education.”*

“*Yep—yeah it was easy to understand. It was actually repetitive, it was so repetitive—you got sick of hearing the same thing.”*

However, some participants felt the information confirmed what they already knew rather than providing new information.

“*I thought I had more information than what the messaging gave. The messaging gave me less than what I knew”*

“*It was just backing up what I already knew, and why we left. It was just clarification—we'd left already, and yes, it was the right thing to do. Some people thought we were overreacting. But until you've got a three-year-old that can't breathe!*”

“*It just sort of proved what we already knew. You couldn't breathe. You couldn't breathe in the house, you couldn't breathe out of the house.”*

“*We would have left anyway. We could tell, that it was quite sickening. We didn't go outside. That period of two weeks it pretty much changed the way we did anything”*

Conversely, some participants recalled advice contradictory to agency messaging, such as directions to wear masks:

“*On the radio, people were saying to shut all the doors and things, but it was pretty practical stuff that you'd kind of do anyway. You know, shut the windows and doors, and if you're outside and its really bad, wear a mask.”*

#### Desire for More Detailed Information

The focus on highly susceptible groups in the public health advice resulted in uncertainty or confusion about the required actions for those outside of those groups. Participants who were not in the nominated risk groups felt as though they acted against the official guidance or that this information was not applicable to their situation:

“*If I listened to the messaging, I would've thought that the smoke didn't impact on me. I'm not part of that group that's in danger. I basically left because I could tell that it had an impact on my own physical health … regardless of the guidelines”*

“*Well, it was mostly directed towards people with certain health conditions that it would be really dangerous to. I don't have any of those diseases myself, so I didn't really think it was relevant.”*

One identified gap in the public advisories were details on the likely short-term and long-term health effects from exposure to this level of smoke:

“*I don't think there was really any information on how the smoke could affect your health. There wasn't really any information. There was just warnings to leave if you have asthma or respiratory—but there wasn't any concrete information about what the effects of the smoke would be.”*

“*The ABC had every 15 min the fire reports which I listened to all the time. There was the website that was updated constantly which I looked at several times a day—the TasFires page … Yeah, that was really good. The ABC—really good. The public meetings were really good. The evacuation center was good. All of that was good. I just think there wasn't enough information the actual health problems that the smoke might cause your health.”*

Had such advice been available, this may have better informed participants on the benefits of protective action to facilitate more considered decision-making and self-efficacy.

“*For example, young children—what do you mean by young children? … if we don't leave, is it something acute that we're risking, or is it some more permanent damage in the long term? So, it's sort of obvious if you've a lung condition and its smoky, it's best not to be here. But is the message ‘it could trigger something permanent,’ you know? So, I think being as specific as they can be is helpful. And support people in those decisions. Because it's hard to leave, it's really hard to leave. It's hard to make that call*.”

#### Some Advice Not Timely or Practical

Commonly, participants had already lived through smoky conditions before they heard public health messaging regarding the smoke.

“*The information about the smoke—the fire information was great—the information about the smoke was too slow, too late.”*

“*I think it was easy to understand, I just think it was too late. I think it just took a really long time to actually get any messages out around how bad it was.”*

“*And then they started saying, ‘no one should go out and do exertional activity outside’ and I thought ‘that's a bit bloody late!’ I've just spent three days outside at [an animal sanctuary] running around with the dogs, you know, helping put up sprinklers—all that sort of stuff.”*

“*The health advice was received right near the end of the bushfire. And it was very minimal. Basically, the advice consisted of ‘if you're concerned, leave’ was the advice. Which is not very practical. Obviously, people who had chronic lung or cardiac issues, that makes a lot of sense. But for people who aren't chronically ill, that doesn't make a lot of sense.”*

However, the perceived delay in public health advisories may have resulted because the available information was not widely promoted until relatively late in the event.

“*I think if you're concerned about air quality, there was already information out there. It just wasn't pushed by the fire authorities at that time.”*

Perceived limitations in their effectiveness or practicability were barriers to adopting recommended actions. For example, very leaky homes cannot be easily sealed to protect against smoke, and the advice to relocate was not practical for many people.

“*But the house doesn't keep the smoke out, we actually had to section off certain areas of the house to be able to sleep.”*

“*They did talk about if you had air-conditioners, they did talk about making sure it was recycle rather than getting the air from outside. But we don't have one anyway, so that's why I didn't take much notice of it (laugh). And yeah, windows and doors shut, but we're such a drafty house anyway, it wasn't making a difference to us.”*

“*On Facebook some groups were saying, ‘oh you can go to the evacuation center’ … I don't think there was enough adequate information given when it was necessary at the very beginning, with options on where to go, and what to do that was satisfactory for a person to deal with it. Running away to sit in a smoke-free room in [an evacuation center] for 2 or 3 hours with oxygen is not a good solution. There are thousands of people here.”*

“*Well apparently the Kingborough one was open for a day then they closed it because no one used it. And I think that was just too far away to be useful.”*

“*I didn't want to go to a refuge, I preferred to go to Hobart in an AirBnB, I didn't think I'd be comfortable in a refuge.”*

A couple from the same household discussed the utility of the smoke refuges for smoke-affected communities and how delays may have resulted in complacency:

A: “So, both of those were advertised through ABC radio and Facebook, and I'm pretty sure there were some posters up in Cygnet about the Kingston evacuation center for people who were smoke-affected.”

B: “But I think hardly, that was set up so late down the track that some people thought ‘oh, well we've been in it for this long’.”

A: “It wasn't straight away, it was a week into it that the Kingston thing started …”

#### Understanding the Meaning of Air-Quality Data

In Tasmania, real-time information about the concentration of fine particulate matter (PM_2.5_) is continuously available from a network of 34 Environmental Protection Authority (EPA) air quality monitoring stations which is shared by the Department of Health and on the smartphone app *AirRater*. International sites also harvest this data without discerning if it is based on hourly or 24-hourly averages and convert it to an air quality index using a formula that is different from that used in Australia. Despite common use or discussion of air quality measures among participants (12 of 24 participants mentioned measures of PM_2.5_), there were difficulties in contextualizing and discerning the reported data:

“*You pop in something like air quality index for Tasmania, and you'd get global one and the EPA one. And the global one seemed pretty alright, but it had a lag. Whereas the EPA one didn't, so working out which one was more accurate was a bit tricky. I assume they use the same data, but the global one might've just taken a bit longer to update.”*

“*Although there was a lot of messages going around on social media on the numbers. You know, what the counts were. But you had no idea what any of them meant. It said 500: well, what's good and what's bad?”*

“*It was almost scare mongering people. And you know, there was a lot of over-reactions, and stuff and it's like ‘oh god’. They don't even know what it means, but they're saying it's so high, its 5 times or 10 times than it normally is, and yeah … it means nothing to me.”*

“*So, if there was a really simple scale on the side that said'this is the range we're aiming for, and this is where you are' that would've been really helpful. But whether the EPA thought to have something like that, because usually we're operating right down, you know … and actually it was a radio interview that explained that best.”*

Participants also indicated they were using a non-government website which collates PM_2.5_ levels worldwide, and that this was more interpretable than the local real-time monitoring system:

“*There was a map of the world, basically. And I'd zoom in on Tassie, and I was watching that daily, and checking to see if there was anywhere else in the world that was worse than us. And for a while, there wasn't … It was very clear on the site that I looked at. So, anything under 50 was good, in the green, and that was basically what I was looking for (laughs) and wherever the green was, that was where I was heading. Or the lower number. But yeah, for the most part we were in the bright red, which was the hazardous. Each of the stages they had very clear meanings behind it. This could affect people with asthma … the higher it went, the more it said it would affect everyone. You know, this is hazardous, you shouldn't be in it for long periods.”*

“*I had a chart to show what healthy was, and it goes up by color. But I think a lot of people had no idea, really.”*

#### Managing the Two Related Hazards of Fire and Smoke

Participants contrasted the smoke and fire hazards during the wildfire event, noting that their previous experiences with smoke during prescribed burns contributed to some indifference toward the smoke:

“*I think everyone just about thinks about the fire. And, yes, I did too. I'm a nurse, I've cared for burns patients, the heat does that. It's awful. To me, that's the worst death. So, I never want to be part of that. But the smoke? You see so many smoke plumes from the burn offs down here as well. It lends well to complacency.”*

However, it was indicated that the importance of fire and smoke hazards were not clearly distinguished, diminishing the perceived need to adopt protective behaviors against the smoke hazard. There was reluctance to divert attention or resources away from dealing with the fires to the lesser hazard of smoke:

“*I don't know if it was actually really used, but there could've been that communication that it's okay to go to an evacuation center just for smoke rather than strictly for fire. That would've been good. And telling people that it's okay to do that. Because I think some people would've felt a bit silly doing that.”*

“*I think getting it a more, maybe, differentiated between the two. Having really distinct sorts of risks coming from the same source, but they're two different risks and two different hazards, and approaching them from different ways, so then you didn't feel as though you were taking resources away from the fire, potential fire victims. Because they're different hazards and one seems much more life-threatening than the other.”*

“*I think the warnings, there should've been more. I know that there's a fire going on, and a danger, a threat to life, and that is probably more important—but I really feel like there's going to be long term health implications now for people such as myself, and I think that that probably needed to be addressed as serious a nature as the fire, and much sooner.”*

“*… if you weren't affected by the fire, you didn't want to make a whoohaa about the smoke when there's people whose lives and homes and properties are at risk. So, I guess it was like all these people are getting affected, so you feel like you just have to suck it up.”*

Finally, fires not only complicated the agency response, they were also a competing priority for community members who tolerated the smoky conditions to prepare their homes or protect animals from the fire. The actions required to protect households from the fire threat (such as clearing shrubbery and other fuel off properties) directly contradicted smoke advice of staying indoors or relocating away from smoke-affected areas:

“*I had to clear that paddock of grass because we slashed it and left it, because if we had an embers coming, it was going light it up.”*

### Perceptions of Portable HEPA Cleaners

Most (20 of 24) householders stated they would use air cleaners again. The smoke period ended abruptly mid-way through the study and several participants did not get the chance to evaluate them during smoky conditions. Only one household indicated they did not perceive any benefit to the conditions within their home; reasoning their home is well-sealed and was never significantly smoky inside. Most households indicated that the air cleaners were unobtrusive and easily incorporated with the homes. Notably, two households were already planning on purchasing an air cleaner while another two had already purchased an air cleaner.

There were different rationales provided for wanting to use air cleaners. One motivating factor was avoidance of the symptoms caused by smoke:

“*I think I probably would, because I was quite disturbed how it made me feel, and I'm a pretty healthy person. And to find that I experienced the discomfort that I did was quite alarming. So, if they were readily available, yes I would.”*

“*I think it's a good precaution, and it did seem like the air inside the house was noticeably fresher. I just don't think it would hurt.”*

Another motivating factor for householders was feeling reassured they were taking as many precautions as they could to protect themselves and their families from the smoke. Others commented they perceived benefits of air cleaners outside of wildfire events, such as during prescribed burns or to mitigate the effects of airborne pollen:

“*I'd consider using it most of the time, especially when there are pollens around because my asthma is affected by that. So yeah, at certain times during the year there are things happening that aggravate it. I do wonder if it would be better in my bedroom, if that would be a better place to have it.”*

“*I think I would still use it, if it was proven to be effective, I think my health would be worth it”*

Similarly, costs to purchase and to power the air cleaners were another key consideration.

“*Yeah, yeah I would [buy an air cleaner] if it was found to be effective, I think it I would. And if it was at a good price point too.”*

“*Depends on the power usage, I suppose …”*

## Discussion

In keeping with previous studies, we found that living through smoky periods was generally a negative experience, and that social media was a central method for receiving information (13, 15). A range of themes emerged from detailed feedback on the public health messaging relating to smoke and provided some key lessons for improving public health practice. These included the perceived lack of timeliness and practicality of some of the information, the desire for more detailed information about health risks and how these related to differing severity of air pollution, and the tension between messaging about the simultaneous fire and smoke hazards in different locations across affected areas. Finally, the HEPA cleaners were generally well-accepted and perceived to be a potentially practical intervention.

The strong theme of the central role of social media during the wildfire and smoke episode was consistent with previous studies. During natural disasters, social media platforms have been demonstrated to enhance public dialogue and the distribution of information in a range of circumstances and settings (24–27). The key advantages include timeliness of information from trusted and valued agencies, such as government, maintenance of community connectedness and enabling community resilience (20, 27, 28). For example, during floods in Queensland, Australia, social media emerged as an important mechanism for government agencies to disseminate information among affected communities (24). However, social media does not reach everyone, and is also a forum for sharing opinion that could either support or undermine the public health response (27). An example of this was the sharing of air quality data from many unofficial sources, accompanied by personal interpretations, which appeared to fill a void in the information available from the local response agencies. Our study found community members used a wide range of information sources in addition to social media but not everyone accessed the available information electronically. This suggests that frequent messages on social media should be central to public health responses, but not be limited to this medium.

The overall good recall of the public health messages about smoke was consistent with previous studies (19, 29). A telephone survey of 389 people following a prolonged smoke event in New South Wales, Australia found 74% of respondents were aware of public health advisories with 57% of these people changing their behavior due to those advisories (13). Kolbe and Gilchrist (13) also identified the most common action taken was reducing outdoor activities across both groups: those who had heard advisories (52%) and those who hadn't (62%). Our study indicated there was good recall of the main messages which were focused on people in higher risk groups, however, participants had difficulty discerning the sources of the communications, and the responses to the advisories varied. Timing was an important contributor to whether participants changed their behavior as a direct response of the official public health messaging, as many participants indicated they adopted protective behaviors prior to receiving any official messaging. Furthermore, in providing public health advisories there is a trade-off between providing simple advice and providing more detailed information, with increased risk of reduced understanding of the information as the complexity of the message increases (29). Overall, many participants in this study would have appreciated more information, especially about risks to people who were not in the named higher-risk groups, potential risk of long-term heath harms, and clearer information about the risk associated with differing severities of smoke pollution. This highlights the need for public messaging to be targeted to meet the needs of diverse audiences (14, 30).

When severe air pollution affects entire communities there is only a limited suite of possible interventions and actions available to individuals to protect their health. Skepticism about the effectiveness, practicality and timing of some recommended actions emerged as a theme in this study. This was not unreasonable given that much of the routine advice provided to the public has a patchy evidence base, especially for Australian settings (31). Many older houses are not well-sealed, and even with closed doors and windows, indoor and outdoor particle concentrations can equilibrate relatively quickly (32). There is some evidence that closing doors and windows can slow indoor penetration of outdoor smoke, but this becomes less effective the longer the duration of the pollution episode (32). Indeed, in the absence of additional indoor air filtering, there is very limited evidence to show that closing doors and windows reduces the health impacts associated with periods of high outdoor air pollution (31). Finally, the use of HEPA cleaners was generally well-accepted by participants during this study, with key motivators for use being to reduce personal symptoms and reassurance. As the participants in this study were all part of a trial of portable HEPA cleaners a favorable attitude is not surprising. Previous studies have found HEPA cleaners to be effective in reducing indoor concentrations of wildfire smoke (33, 34). Observations from this study suggest that more research is needed to improve and refine the evidence base for a range of interventions for health protection during severe air pollution events.

Timing is often reported as being a core consideration to effective crisis communication, and delays in communication have previously been found to be an important contributor to public misunderstanding, as demonstrated in a case study of a smoke event which resulted from coal mine fire in Victoria, Australia (35). Several participants in this study relocated to reduce their exposure to smoke and many did so before the advice for this course of action was provided, and commented that the advice came too late, after the worst of the smoke had passed. Timing of advice is a major challenge for authorities during wildfire smoke events because it is difficult to forecast smoke impacts several days ahead as conditions can change very quickly (36). Krstic and Henderson (37) illustrated this problem when they evaluated satellite data to assess smoke exposure before and after 41 community evacuations for poor air quality associated with wildfires and found that air pollution had peaked prior to the evacuation in around half of all cases. This is noteworthy, as the benefit of relocation must be balanced against the potential harms, especially for higher risk groups, such as older people (38).

A major theme that emerged from the interviews was the tension and confusion that arose because public advice relating to the wildfire episode and resulting smoke were issued from a range of organizations. For example, Tasmania Fire Service continually updated messaging about: specific fires and the associated threat level for specific communities, community meetings, evacuation centers, travel restrictions, phone network outages, and information from other agencies about air pollution and health. This information saturated traditional news media and social media networks. Thus, public health messaging about smoke competed with many other public messages, some of which related to immediate and potentially life-threatening aspects of the emergency. This created a tension for many participants as they were not aware of the smoke information until later in the wildfire episode, or they felt they were over-reacting to their smoke experiences because the fire emergency across the river at the firegrounds was being more extensively covered in media. Some participants suggested that smoke information should be an integral part of wildfire communications with a single place to go with links to all relevant information easily accessible from there. Well-integrated links between wildfire and smoke resources, with easy access to information about air pollution and its associated health risks could improve the effectiveness of smoke-related public health messaging and increase self-efficacy and appropriate risk mitigation behaviors (30, 39, 40). This is an avenue to improve the coordination of public messaging during concurrent wildfire and smoke events. Although the health effects of smoke are small in comparison to a direct encounter with flames, smoke reaches far more people and can ultimately cause many more admissions to hospital and premature deaths than the fire fronts themselves. For example in Sydney, Australia, 197 deaths were attributable to smoke from vegetation fires from 2001 to 2013 (41), while five direct fire-related deaths were reported for the state during the same period, and 77 direct fire-related deaths were reported for the century between 1901 and 2011 (42). While communication about immediately life-threatening hazards clearly should be prioritized during wildfire events, this should not be a reason to disregard the wider-scale impacts of smoke on population health.

We acknowledge limitations to our study. The concurrent emergency imposed several practical constraints. It needed to be designed, approved and implemented within a 48-h period and was therefore necessarily small in scale, with a limited number of eligible locations, and restricted to people willing to participate in a study of portable HEPA cleaners. For these reasons the experiences and perceptions from the full diversity of people living in the affected communities was unlikely to have been be captured in the study. A strength of our study was that it took place during a wildfire emergency, which enabled researchers to obtain detailed and rich information while the situation and topics of discussion were highly relevant to participants, rather than many months later which has been more typical of previous reports of post-fire communication evaluations (13, 29, 35). The detailed interviews enabled the collection of a greater depth and richness of information than that which would be possible from a broad scale retrospective survey.

Our study has highlighted some of the special challenges in risk communication about wildfire smoke and barriers community members face in understanding and responding to the public health hazard. Many participants were concerned of the health impacts of smoke during the 2019 wildfire event in Tasmania. While public health messaging on smoke was widely shared and understood, with social media playing a central role in this, some participants expressed concerns about the timeliness and effectiveness of the interventions, and some would have appreciated more detailed information about the health risks from smoke to better inform behavior change. Many participants identified confusion with the multiple public messages and actions relating to the more seriously perceived threat from the firegrounds. We concluded that public messaging about smoke and health should continue to use multiple avenues of communication with a focus of simple messages through social media, and that it would be more effective in informing communities if integrated with wildfire-related public messaging and clear links to more detailed information about air quality smoke and health for those seeking further information.

## Data Availability Statement

The datasets generated for this study are available on request to the corresponding author.

## Ethics Statement

The studies involving human participants were reviewed and approved by Human Research Ethics Committee of the University of Tasmania. The participants provided their written informed consent to participate in this study.

## Author Contributions

MM conducted the interviews, data analysis, and led the writing of the paper. FJ led the study with AW, SC, KG, SM, and MV developed the study protocols, supervised the analysis, and contributed to the manuscript. NB-A assisted with the data collection, generated the figures, and contributed to the manuscript. All authors contributed to the article and approved the submitted version.

## Conflict of Interest

The authors declare that the research was conducted in the absence of any commercial or financial relationships that could be construed as a potential conflict of interest.
